# Nearly Complete Genome Sequence of a Human Norovirus GII.P17-GII.17 Strain Isolated from Brazil in 2015

**DOI:** 10.1128/MRA.01376-18

**Published:** 2019-01-31

**Authors:** Cristina Santiso-Bellón, Azahara Fuentes-Trillo, Juliana da Silva Ribeiro de Andrade, Carolina Monzó, Susana Vila-Vicent, Roberto Gozalbo Rovira, Javier Buesa, Felipe J. Chaves, Marize Pereira Miagostovich, Jesús Rodríguez-Díaz

**Affiliations:** a Department of Microbiology, School of Medicine, University of Valencia, Valencia, Spain; b Department of Genomics and Genetic Diagnosis Unit, Hospital Clínico Research Foundation (INCLIVA), Valencia, Spain; c Laboratory of Comparative and Environmental Virology, Oswaldo Cruz Institute, Rio de Janeiro, RJ, Brazil; d CIBER of Diabetes and Associated Metabolic Diseases (CIBERDEM), Madrid, Spain; KU Leuven

## Abstract

Human noroviruses are the most common cause of nonbacterial acute gastroenteritis worldwide. We report here the nearly complete genome sequence (7,551 nucleotides) of a human norovirus GII.P17-GII.17 strain detected in July 2015 in the stool sample from an adult with acute gastroenteritis in Brazil.

## ANNOUNCEMENT

Human noroviruses are the most common cause of nonbacterial acute gastroenteritis (AGE) and the leading cause of sporadic cases and outbreaks in children and adults worldwide (1, 2). The Norovirus genus belongs to the *Caliciviridae* family and is classified into six distinct genogroups (GI to GVI), which are further subdivided into different genotypes (3). Strains belonging to the same genotype share a sequence similarity of higher than 85% (4). Only genogroups I (GI), II (GII), and IV (GIV) have been identified in humans. GII is the most diverse genogroup, being subdivided into more than 20 genotypes and currently with a wide distribution, playing a major role in the etiology of AGE. For decades, the most prevalent genotype worldwide has been GII.4. In the winter of 2014 to 2015, the GII.17 genotype replaced the circulating GII.4 genotype in Asia, the GII.4 Sydney_2012 variant (5, 6). Although the origin of GII.17 strains is still largely unknown, the GII.17 Kawasaki_2014 variant caused large AGE outbreaks in China, Japan, and South Korea (6–9) and sporadic infections in Hong Kong, Taiwan (10), the United States (11), Italy (12), Romania (13), and Australia. In Latin America, this emergent strain was detected in AGE outbreaks and sporadic cases in Brazil (14, 15) and in sporadic cases in Argentina (16).

We report here the complete genome sequence of a human norovirus GII.P17-GII.17 strain detected in feces from an adult with AGE in July 2015 in Brazil.

Viral RNA was extracted from 140 µl of a 20% fecal suspension in phosphate-buffered saline (PBS), using the QIAamp viral RNA minikit (Qiagen, Hilden, Germany) with the QIAcube automatized system (Qiagen). Total RNA was sent to Macrogen, Inc., for library construction using a TruSeq mRNA library prep kit (Illumina) and sequencing on a HiSeq platform (Illumina). Data were analyzed using different open-source bioinformatics software programs. A total of 68,795,022 paired-end 100-bp-long reads were obtained. Raw read quality control was assessed with FastQC version 0.11.5 (17). Reads were quality trimmed using seqtk trimfq (version 1.2-r101-dirty) (18). One contig covering the Norovirus genome sequence (7,551 nucleotides [nt]) was assembled using SPAdes –meta (version 3.11.1) (19), with autoadjusted k-mer lengths of 21 nt, 33 nt, and 55 nt, containing an average depth of coverage of 455.09× (35,098 reads). Only 0.051% of the reads (35,098 of 68,795,022 reads) belonged to the Norovirus genome, and the final coverage of the genome was higher than 99% (7,551/7,569 nt).

The genome sequence (7,551 nt) contains three open reading frames (ORFs). ORF1 is incomplete and extends from nucleotides 1 to 5095 (1,697 amino acids [aa]), ORF2 extends from nucleotides 5076 to 6698 (1623 nt and 540 aa), and ORF3 starts at nucleotide 6698 and finishes at nucleotide 7477 (780 nt and 259 aa). The genotype was assigned using the Norovirus Genotyping tool (20). Afterwards, BLASTn was run, and it was found that this sequence has 99.52% (7,515/7,551) and 99.50% (7,513/7,551) nucleotide similarity with two GII.17 strains (GenBank accession numbers KT380915 and KP998539, respectively). The contig obtained lacked the first 18 nucleotides compared to those sequences (GTGAATGAAGATGGCGTC). SSPACE version 3.0 (21) and AlignGraph (22) were used to try to extend the assembled contig. Reads were mapped using the BWA-MEM version 0.7.15-r1140 software (23) against the identified most closely related reference genome sequences. The first 18 nucleotides at the 5′ end were still not found.

Our study describes a nearly complete genome sequence of a Norovirus GII.P17-GII.17 genotype strain providing additional information relevant to be used as a reference for phylogenetic and evolutionary studies.

### Data availability.

The assigned accession number for the present norovirus genome in GenBank is MH997861. The ORFs were given the GenBank accession numbers AYF59857 (ORF1), AYF59855 (ORF2), and AYF59856 (ORF3). The sequence data are available in the Sequence Read Archive (SRA) under BioProject number PRJNA497363.
